# Inhibition of microglial receptor‐interacting protein kinase 1 ameliorates neuroinflammation following cerebral ischaemic stroke

**DOI:** 10.1111/jcmm.15820

**Published:** 2020-09-29

**Authors:** Yang Jiao, Jianjian Wang, Huixue Zhang, Yuze Cao, Yang Qu, Siyu Huang, Xiaotong Kong, Chang Song, Jie Li, Qian Li, Heping Ma, Xiaoyu Lu, Lihua Wang

**Affiliations:** ^1^ Department of Neurology The Second Affiliated Hospital Harbin Medical University Harbin China; ^2^ Department of Neurology Peking Union Medical College Hospital Chinese Academy of Medical Sciences Beijing China; ^3^ Department of Pharmacology (The State‐Province Key Laboratories of Biomedicine‐Pharmaceutics of China) Harbin Medical University Harbin China; ^4^ Department of Physiology Emory University School of Medicine Atlanta GA USA

**Keywords:** ischaemic stroke, microglia, NLRP3 inflammasome, rhTrx‐1, RIPK1

## Abstract

Microglia are rapidly activated following ischaemic stroke and participate in the induction of neuroinflammation, which exacerbates the injury of ischaemic stroke. However, the mechanisms regulating ischaemic microglia remain unclear. In the present study, middle cerebral artery occlusion and oxygen and glucose deprivation models were established for in vivo and vitro monitoring of experimental stroke. We applied recombinant human thioredoxin‐1 (rhTrx‐1) and Necrostatin‐1 (Nec‐1, inhibitor of RIPK1) to examine the role of receptor‐interacting protein kinase 1 (RIPK1) in the development of inflammation in ischaemic microglia via explored the inflammatory responses and the associated mechanisms. Molecular docking results indicated that rhTrx‐1 could directly bind to RIPK1. In vivo and vitro data revealed that rhTrx‐1 reduced necroptosis, mitochondrial membrane potential damage, reactive oxygen species accumulation and NLR Family, pyrin domain‐containing 3 protein (NLRP3) inflammasome activation and regulated the microglial M1/M2 phenotypic changes by inhibiting RIPK1 expression in ischaemic microglia. Consistent with these findings, further in vivo experiments revealed that rhTrx‐1 treatment attenuated cerebral ischaemic injury by inhibiting the inflammatory response. Our data demonstrated the role of RIPK1 in microglia‐induced neuroinflammation following cerebral ischaemia. Administration of rhTrx‐1 provides neuroprotection in ischaemic stroke‐induced microglial neuroinflammation by inhibiting RIPK1 expression.

## 1 INTRODUCTION

The global incidence of ischaemic stroke has been increasing annually and has become the leading cause of disability and death for adults worldwide.^1^ The incidence of the neuroinflammatory response that is induced within minutes to hours after the onset of acute ischaemic stroke mediates the damage in the brain tissues and cells following ischaemia and is considered an important cause of acute ischaemic injury.^2^, ^3^ Microglia are important immunocompetent cells in the central nervous system.^4^ Following the incidence of ischaemic stroke, microglia are rapidly activated into different phenotypes and participate in the damage or repair of ischaemic brain tissue by regulating the occurrence of neuroinflammatory reactions.^5^, ^6^ The molecular mechanisms that regulate microglial activation are still unclear. Exploration of these mechanisms is beneficial to the development of effective drugs for the treatment of cerebral ischaemic injury.^7^, ^8^

RIPK1 plays an important role in mediating the inflammatory response.^9^ RIPK1 can initiate necroptosis by activating RIPK3, which then induces necrosis‐like changes in cells and releases inflammatory factors.^10^ In addition to relying on the necroptotic pathway to release inflammatory factors, RIPK1 can also release inflammatory factors by driving NLRP3 inflammasome activation.^11^ Intracerebroventricular injections of Nec‐1 (a specific inhibitor of RIPK1) can prematurely reduce the volume of cerebral infarction more effectively compared with the therapeutic effects noted by the inhibition of apoptosis.^12^, ^13^ This evidence implied that treatment with targeted inhibition of RIPK1 could widen the time window of treatment in the acute phase of cerebral infarction. Current studies suggested that neurons undergoing necroptosis could recruit microglia leading to secondary injury by releasing inflammatory factors.^14^ However, the role of RIPK1 in driving the inflammatory response in microglia following cerebral ischaemia is still unclear. Therefore, further investigations are required.

The inhibition of the inflammatory response following acute phase reperfusion plays an important role in the treatment of cerebral infarction.^2^, ^3^ Thioredoxin‐1 (Trx‐1) is a small molecular weight hydrogen donor widely present in cells and exhibits a variety of biological functions, such as antioxidant and anti‐inflammatory activities.^15^, ^16^ Previous studies have shown that overexpression of Trx‐1 provides effective neuroprotective effects on the brain tissue of middle cerebral artery occlusion (MCAO) mice, while inhibition of Trx‐1 exacerbates the neuroinflammatory response in MCAO mice.^17^, ^18^, ^19^ These findings provide a strong scientific basis for the clinical translational application of Trx‐1. Recombinant human thioredoxin‐1 (rhTrx‐1) is a compound extracted and synthesized from *Escherichia coli*. Current studies have shown that the use of rhTrx‐1 treatment could reduce cerebral infarction volume and improve neurological deficits in MCAO mice. In addition, rhTrx‐1 treatment could alleviate ischaemia‐induced neuron injury.^20^, ^21^, ^22^ Nevertheless, the protective and regulatory effects of rhTrx‐1 on microglia following cerebral ischaemia remain unclear. Lee et al reported that rhTrx‐1 pre‐treatment could prevent N‐acetyl‐p‐aminophenol induced mouse liver injury by eliminating reactive oxygen species (ROS) and inhibiting the up‐regulation of RIP‐3 levels (downstream protein of RIPK1).^23^ However, the effects of rhTrx‐1 on RIPK1 levels are unclear. Based on these findings, the present study aimed to investigate the therapeutic potential of rhTrx‐1 in inhibiting RIPK1‐driven neuroinflammation in microglia following cerebral ischaemia.

## 2 MATERIALS AND METHODS

### 2.1 Chemical materials

Pentobarbital sodium was safeguarded in Harbin Medical Pharmacological Laboratory. BSA and rhTrx‐1 were purchased from R&D System. Penicillin/streptomycin solution, Hoechst and 2% TTC dyeing solution were purchased from Solarbio. ROS and Necrostatin‐1 was purchased from Sigma. Annexin V‐PE/7AAD assay was purchased from BD Biosciences. All the ELISA kits were purchased from Cusabio. Dapi dyeing solution and JC‐1 kit were purchased from Beyotime. DMEM, foetal bovine serum (FBS) and MEM/EBSS were purchased from Hyclone. The primary antibodies used for immunofluorescence staining: Rabbit polyclonal anti‐Iba‐1 (Wako), Goat polyclonal anti‐Iba‐1 (Abcam), Mouse monoclonal anti‐RIPK1 (Santa cruz), Goat polyclonal anti‐CD206 (R&D System) and Rabbit polyclonal anti‐CD16 (Bioss). All the second antibodies used for immunofluorescence staining were purchased from Abcam company. The primary antibodies used for Western blot analysis: anti‐RIPK1, anti‐RIPK3, anti‐MLKL, anti‐pMLKL and anti‐CCL2 (Abcam); anti‐NLRP3, anti‐ASC, anti‐caspase‐1, anti‐caspase‐3 and anti‐β‐actin (ABclonal); anti‐MMP‐9 (R&D System); and anti‐CD16 (Bioss). The second antibodies used or Western blot analysis was Alexa Fluor 800‐conjugated Goat‐anti rabbit (LI‐COR).

### 2.2 MCAO model and drug administration

All animal experiments were conducted in accordance with the Animal Care and Use Committee of the Second Affiliated Hospital of Harbin Medical University. Male C57 BL/6 mice (7‐8 weeks, 20‐25 g) were treated for 60 minutes to induce MCAO as described previously with some modifications.^24^ Briefly, mice were randomly divided into the three following groups: Sham, MCAO and MCAO + rhTrx‐1 group. All mice were anaesthetized with pentobarbital sodium prior to MCAO induction. During the operation, the animal body temperature was kept at 37°C. The establishment of MCAO required the exposure and separation of the common carotid artery (CCA), external carotid artery (ECA) and internal carotid artery (ICA). The CCA was clipped, and ligation was performed at the ECA with a small incision. Through the incision, the thread embolism was slowly inserted into ICA. Following 60 minutes occlusion, the thread embolism was removed and the reperfusion was carried out. The mice of the Sham group received the same procedure without the embolus. The mice in the MCAO + rhTrx‐1 group received 10 mg/kg rhTrx‐1 by tail vein injection following reperfusion. The control group included mice in the MCAO group that were injected with an equal volume of 0.9% sterile saline. All mice were killed 24 hours following reperfusion for further analysis.

### 2.3 Infarct volume assessment

TTC staining was performed to evaluate the size of cerebral infarction. In brief, the brain was obtained to produce coronal brain sections after the mice were killed. The slices were placed in 2% TTC staining solution and immersed for 10 minutes at 37°C avoiding any cross‐contamination. Following staining, the slices were fixed in 4% paraformaldehyde (PFA) overnight and imaged the following day. Each infarct area was measured using the Image J software.

### 2.4 Neurobehavioral testing

To assess the degree of sensory and motor damage in each group of mice, the Bederson score and corner test were carried out based on previous studies.^25^, ^26^ In order to evaluate the Bederson score, the scores were recorded according to the physical signs of the mice in the tail suspension state as follows: 0 points, normal; 1 point, the contralateral forelimb could not be fully extended; 2 points, the resistance to the thrust of the contralateral forelimb decreased; and 3 points, turning to the contralateral side of the lesion. The evaluation corner test was conducted by placing the mice in the depth of the 30° angle, and the direction of turning was observed. Normal mice turned randomly towards both sides, whereas the mice turned towards the lesion side following cerebral ischaemia.

### 2.5 OGD induction and co‐culture of microglia with neuron cells

Cells were cultured in complete medium and incubated at 37°C in an incubator containing 5% CO_2_. The complete medium was composed of DMEM containing 10% FBS and 1% penicillin/streptomycin solution. Oxygen and glucose deprivation (OGD) was induced by replacement of the cell supernatant with EBSS solution and cell transfer to three gas incubators (37°C, containing 95% N_2_ and 5% CO_2_). For co‐culture, HT22 received culture medium (CM) from BV2 cells under OGD. After cultured for 24 hours, the HT22 cells were collected for the apoptosis ratio detection.^27^ Cells in the Nec‐1 group were treated with Nec‐1 (20 μmol/L) following reoxygenation and in the rhTrx‐1 group were incubated with 5, 10 or 25 μg/mL rhTrx‐1 (dissolved in sterile PBS) for 24 hours following reoxygenation.

### 2.6 Flow cytometry

To detect the apoptotic rate, the intracellular ROS levels, and the concentration of CD86 and CD206, the Annexin V‐PE/7AAD kit and the DCFH‐DA solution were used according to the manufacturer's instructions. The percentage of apoptotic cells was evaluated by double staining with Annexin V‐PE and 7AAD for 15 minutes at room temperature in the dark. To detect the intracellular ROS levels, the cells were harvested and stained with DCFH‐DA solution for 20 minutes in the dark at 37°C. To detect the concentration of CD86 and CD206, cells were incubated with CD86 or CD206 antibodies and finally loaded into the flow cytometer for detection. The cells were analysed using a Beckman CytoFLEX flow cytometer.

### 2.7 Western blot analysis

Protein levels were detected by Western blot analysis. In brief, the cells or brain tissues were lysed in pre‐cooled RIPA buffer and centrifuged to collect the supernatant for protein extraction. The samples were loaded on SDS gels, subjected to electrophoresis and subsequently transferred onto nitrocellulose membranes. Then, the membranes were blocked with 5% BSA or 5% non‐fat dry milk and finally incubated with primary antibodies (anti‐RIPK1, anti‐RIPK3, anti‐MLKL, anti‐pMLKL, anti‐CCL2, anti‐MMP‐9, anti‐NLRP3, anti‐ASC, anti‐caspase‐1, anti‐caspase‐3 and anti‐β‐actin) at 4°C overnight. The following day, the membranes were washed and incubated with Alexa Fluor 800‐conjugated Goat‐anti rabbit antibody at room temperature and then imaged and analysed with the Odyssey system (LI‐COR Biosciences).

### 2.8 Transmission electron microscopy (TEM)

Following collection of the cell precipitates, glutaraldehyde was added slowly for fixation. Ultrathin cell sections (100 nm thick) were prepared using ultramicrotome, mounted on a copper grid and finally stained with uranyl acetate and lead citrate. The sections were observed at an accelerating voltage of 80 kV on a transmission electron microscope (Hitachi H‐7100).

### 2.9 Immunofluorescence staining

Briefly, in vitro immunofluorescence staining was performed with cell fixation in the presence of 4% PFA for 15 minutes and subsequent washing with PBS for three times. Primary antibodies were then added to the cells. Following incubation with anti‐CD206 or anti‐CD16 primary antibodies at 4°C overnight, the cells were incubated with the secondary antibodies (the Alexa Fluor 555‐conjugated donkey‐anti rabbit and Alexa Fluor 488‐conjugated donkey‐goat rabbit) at room temperature for 1 hour and Hoechst was added at the final step. In vivo immunofluorescence was assessed using frozen slices that were washed with PBS for three times and subsequently blocked with 5% BSA at room temperature for 1 hour. The anti‐RIPK1, anti‐CD206, anti‐CD16 and Iba‐1 primary antibodies were incubated with the slices at 4°C overnight. The second day slices were incubated with the secondary antibodies (Alexa Fluor 555‐conjugated donkey‐anti rabbit and Alexa Fluor 488‐conjugated donkey‐anti mouse were used for Iba‐1 with RIPK1 staining; Alexa Fluor 555‐conjugated donkey‐anti rabbit and Alexa Fluor 488‐conjugated donkey‐goat were used for Iba‐1 with CD206 staining; the Alexa Fluor 488‐conjugated donkey‐anti rabbit and Alexa Fluor 555‐conjugated donkey‐goat rabbit were used for Iba‐1 with CD16 staining) at room temperature for 1 hour, and Dapi was added at the final step. The slices and cells were imaged with fluorescence microscopy (ZEISS).

### 2.10 ROS detection

Prior to cell collection, DCFH‐DA was diluted with serum‐free DMEM at a dilution ratio of 1:1000. To detect the intracellular generation of ROS in vitro, the treated cells were incubated with diluted DCFH‐DA solution for 20 minutes in a 37°C incubator protected from light. Following washing three times with serum‐free cell CM, the cells were immediately imaged under a fluorescence microscope (ZEISS) and analysed using the Image J software.

### 2.11 Mitochondrial membrane potential (MMP) assay

The JC‐1 staining kit was used to measure the MMP in vitro according to the manufacturer's instructions. Briefly, the JC‐1 buffer and working solutions were prepared after the cells were subjected to 4 hours OGD and 24 hours reoxygenation conditions. The appropriate volume of the JC‐1 working solution was added to fully cover the cells in place of the cell CM, and subsequently, the cells were cultured at 37°C for 20 minutes. Following washing three times with the JC‐1 buffer solution, the cells were examined immediately using a ZEISS fluorescence microscope.

### 2.12 ELISA

To detect the inflammatory factor levels, the cell supernatant or brain tissues were collected and ELISA was performed according to the manufacturer's instructions. Briefly, 50 μL sample and 100 μL antibody were tested. These reagents were added to the reaction wells, and then, the plate was incubated at 37°C for 60 minutes in the dark. Following incubation with the termination solution, the OD value was measured at 450 nm to obtain the expression levels of TNF‐α, IL‐1β and TGF‐β.

### 2.13 Molecular docking

The structures of rhTrx‐1 and RIPK1 were processed and optimized using the Accelrys Discovery Studio 2016 platform. Protein docking was performed and calculated in the ZDOCK module. The poses with the best scores were selected, and subsequent energy optimization was performed with the RDock program. The interaction between rhTrx‐1 and RIPK1 was analysed using the Analyze Protein Interface module. Finally, Pymol (DeLano Scientific) was used for mapping.

### 2.14 Statistical analysis

The GraphPad Prism (Version 6.0c) software was used for statistical analysis. Student's *t* test and Mann‐Whitney *U* test were used to analysis the differences between two groups. One‐way ANOVA was used to evaluate multiple comparisons statistical significance between more than two groups. The results were presented using mean standard deviation. A *P* < .05 was considered for significant differences.

## 3 RESULTS

### 3.1 RIPK1 is induced in the microglia of MCAO following reperfusion

**Figure 1:**
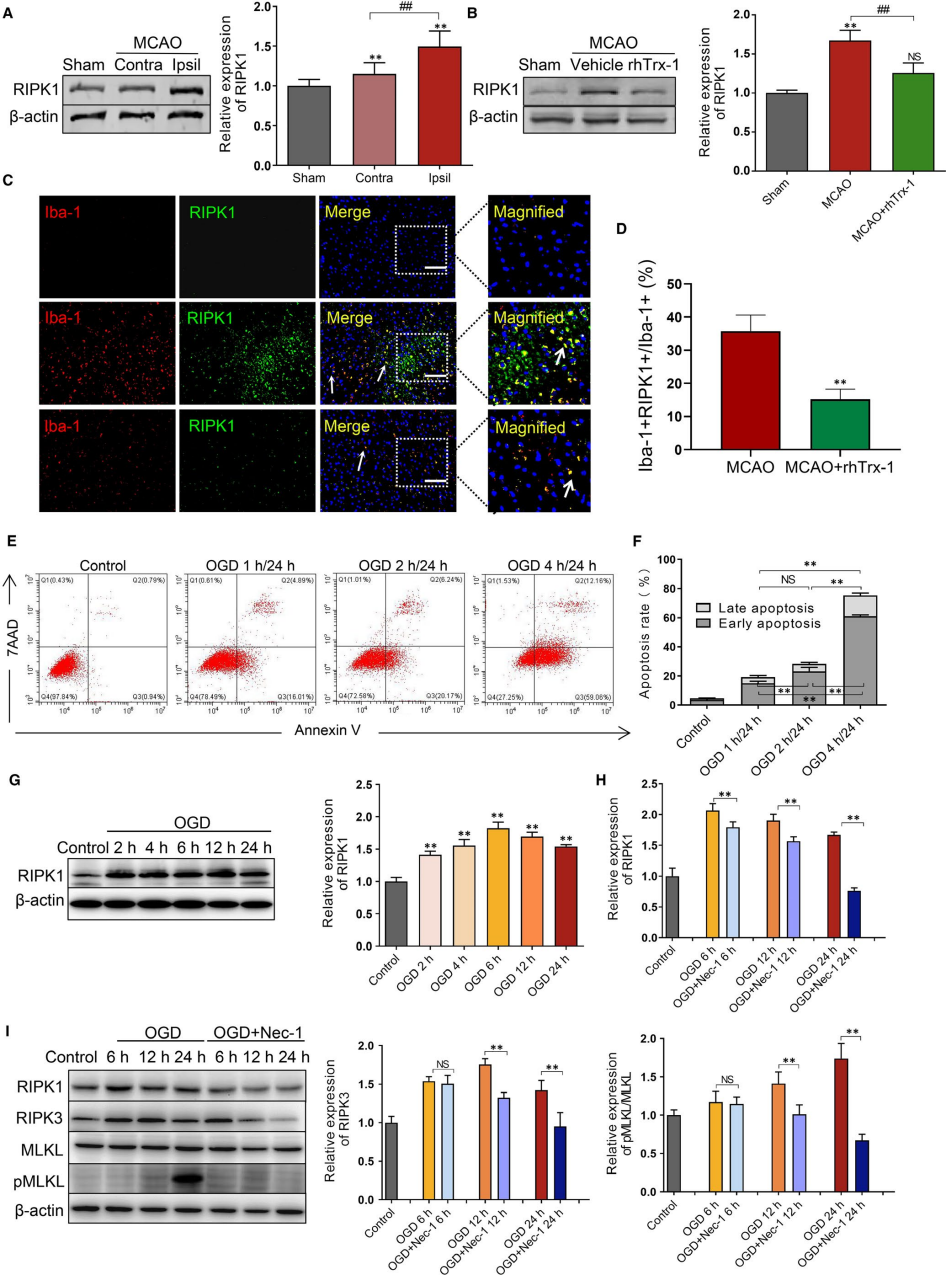
Detection of RIPK1 level and RIPK1‐mediated necroptosis in ischaemic microglia. A, RIPK1 levels were detected and quantificated in the MCAO contralateral (Contra) hemisphere and ipsilateral (ipsil) hemisphere (n = 6 per group). B, The RIPK1 levels were detected and quantificated using Western blot analysis in the MCAO mice following administration of rhTrx‐1 (n = 6 per group). C, Co‐localization fluorescence staining of RIPK1 and microglia at 24 h following reperfusion in Sham, MCAO and rhTrx‐1 group. The scale bar represents 50 μm (n = 5 per group). D, Quantification of RIPK1^+^Iba^+^ cells in microglia. E, The apoptotic rate of microglia at 1, 2 and 4 h of OGD following 24 h reperfusion was detected by flow cytometry using Annexin V/7AAD staining. F, Quantification analysis of flow cytometry (n = 4 per group). G, RIPK1 levels were analysed at 2, 4, 6, 12 and 24 h reperfusion following 4 h of OGD by Western blot. H, I, Necroptosis protein levels with Nec‐1 treatment were examined by Western blot at 24 h reperfusion following 4 h of OGD and quantification data of RIPK1, RIPK3 levels and pMLKL/MLKL level (n = 4 per group). **P < .01; ^##^ *P* < .01; NS, no significance

We applied the MCAO model and examined the expression of RIPK1 in the mouse brain 24 hours after reperfusion. Initially, the samples were removed from the contralateral and ipsilateral hemisphere and were subsequently examined by Western blot analysis. The expression of RIPK1 was significantly increased on the ipsilateral hemisphere compared with that of the contralateral hemisphere (Figure 1A). As researches showed that RIPK1 levels were increased in endothelial cells and astrocytes after MCAO,^28^, ^29^ we also verified that RIPK1 expressed in endothelial cells and astrocytes following MCAO (Figure S1A,B). However, there are no studies on microglia about it. Therefore, to verify the therapeutic effects of rhTrx‐1 in vivo, we measured the RIPK1 levels in the rhTrx‐1 treated MCAO mice. The expression levels of RIPK1 were uniformly inhibited by administration of rhTrx‐1 compared with the untreated MCAO group (Figure 1B). Subsequently, our study aimed to assess the localization of RIPK1 in microglia by double staining of RIPK1 and Iba‐1. The data indicated that RIPK1 expression was noted in microglia following MCAO (Figure 1C), and administration of rhTrx‐1 reduced the microglial RIPK1 following MCAO (Figure 1D).

### 3.2 RIPK1 expression levels following induction of necroptosis of OGD microglia in vitro

To investigate the degree of microglial necroptosis at different OGD time periods, flow cytometry was performed. The results indicated that the number of early and late apoptotic microglia was gradually increased following increased exposure to OGD time periods of treatment (Figure 1E,F). The highest effect was noted at 24 hours of reoxygenation after 4 hours of GOD. Based on this evidence, further investigations of the expression levels of RIPK1 were conducted at different reoxygenation time periods following 4 hours of OGD. The data demonstrated that RIPK1 peaked at 6 hours of reoxygenation (Figure 1G). Furthermore, therapeutic treatment of Nec‐1 restrained RIPK1, RIPK3 and pMLKL/MLKL levels compared with those of the untreated groups (Figure 1H,I). The inhibitory effect was the highest at 24 hours of reoxygenation. Taken together, these results indicated that RIPK1‐mediated necroptosis was augmented in microglia following ischaemia reperfusion conditions.

### 3.3 RhTrx‐1 decreases RIPK1‐induced necroptosis and apoptosis of OGD‐microglia in vitro

**Figure 2:**
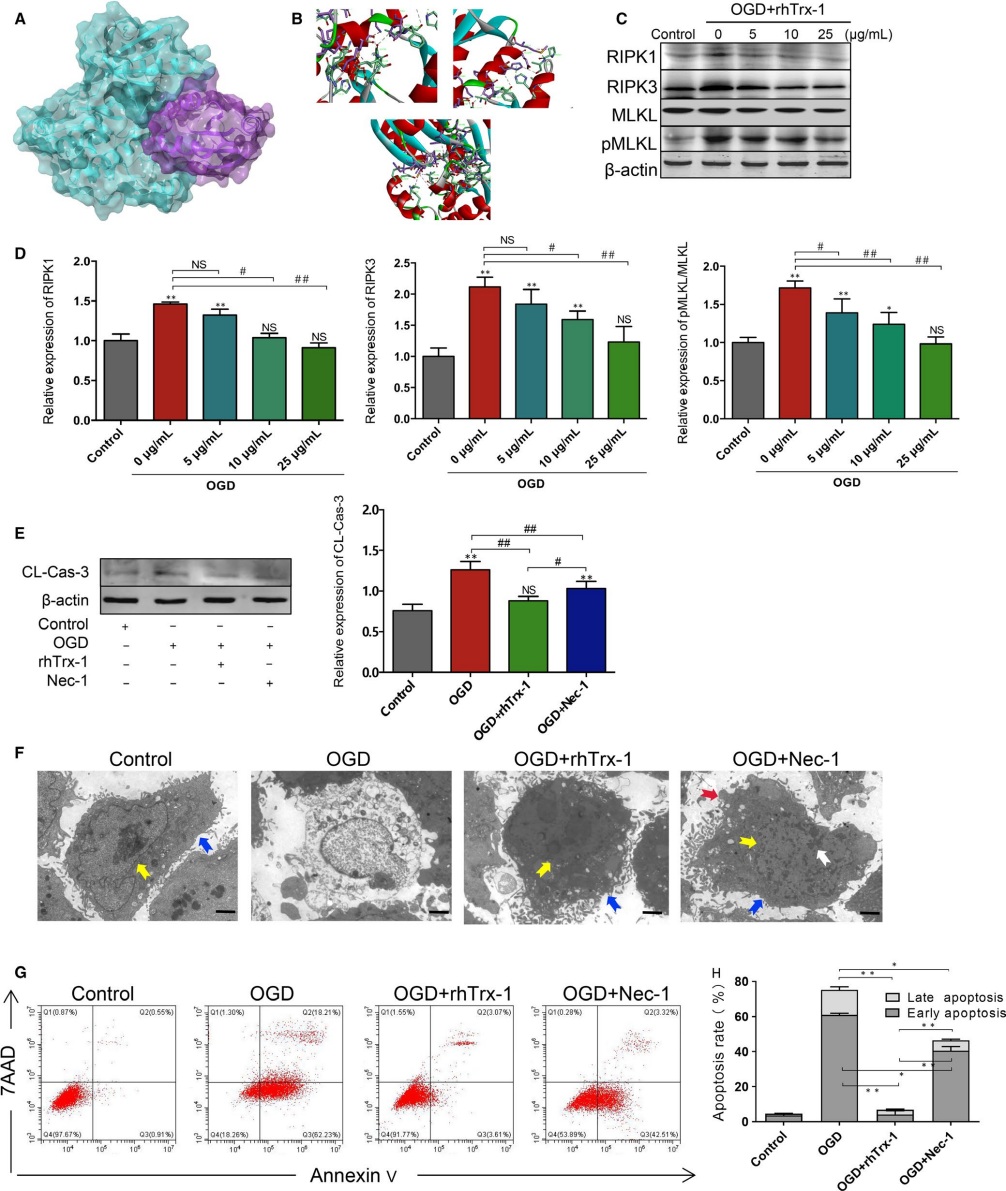
Effects of rhTrx‐1 treatment on necroptosis and apoptosis in vitro. A, Docking result of RIPK1 and rhTrx‐1. Green molecular images represent RIPK1, and purple molecular images represent rhTrx‐1. B, Interface analysis of docking. C, Representative images of the difference dose of rhTrx‐1 treatment on the expression of necroptosis pathway proteins 24 h following OGD. D, Quantification analysis of the protein levels of RIPK1, RIPK3 and pMLKL/MLKL (n = 4 per group). E, Western blot analysis of CL‐Cas‐3 (cleaved‐caspase‐3) levels and quantification data are displayed (n = 4 per group). F, Ultrastructural changes of rhTrx‐1 and Nec‐1 treated cells (Blue arrow = intact cell membrane; yellow arrow = nuclear membrane; white arrow = hyperconcentrated chromatin; red arrow = apoptotic body; Scale bar represents 2 μm). G, Annexin V/7AAD double‐staining images of rhTrx‐1 and Nec‐1 treated cells. H, Quantification analysis of Annexin V/7AAD (n = 4 per group). *P < .05, **P < .01; ^#^ *P* < .05, ^##^ *P* < .01; NS, no significance

To characterize the role of rhTrx‐1, we analysed the binding interaction of rhTrx‐1 and RIPK1 by computational analysis. Docking analysis illustrated six Pi interactions, five hydrogen bonds and one salt bridge demonstrating the interaction between rhTrx‐1 and RIPK1. These results indicated that rhTrx‐1 may direct binding with rhTrx1 RIPK1 (Figure 2A,B). Further detection indicated that the doses of 5, 10 and 25 μg/mL of rhTx‐1 treatment could inhibit the expression levels of RIPK1, RIPK3 and pMLKL/MLKL following 4 hours exposure to OGD conditions and that the inhibitory effect of rhTrx‐1 was more apparent at the dose of 25 μg/mL (Figure 2C,D). According to this evidence, follow‐up experiments were carried out with 25 μg/mL of rhTrx‐1 as the optimal concentration. Besides, as Figure S1C seen, the level of RIPK3 (MFI 15 368 ± 524) in OGD grouped was increased compared with Control group (MFI 8693 ± 247) by flow cytomertry analysis. Treatment with rhTrx‐1 (MFI 10 794 ± 391) and Nec‐1 (MFI 8718 ± 426) led to an decreased level of RIPK3 expression. Subsequent investigation revealed that rhTrx‐1 and Nec‐1 treatment reduced cleaved‐caspase‐3 levels (Figure 2E). Ultrastructure images of TEM indicated that cell membrane incomplete, plasma membrane rupture and other cell death characteristics in OGD group; contraction, nuclear condensation and other apoptotic characteristics and relatively fewer features of cell death in Nec‐1 treatment group; few characterizations of cell death and apoptosis in the rhTrx‐1 treatment group (Figure 2F). In addition, compared to the untreated groups, a lower number of microglia underwent early and late apoptosis in the rhTrx‐1 and Nec‐1 treatment groups following exposure to OGD conditions (Figure 2G,H).

### 3.4 Therapeutic treatment with rhTrx‐1 mitigates OGD‐induced microglial mitochondrial injury and NLRP3 inflammasome activation

**Figure 3:**
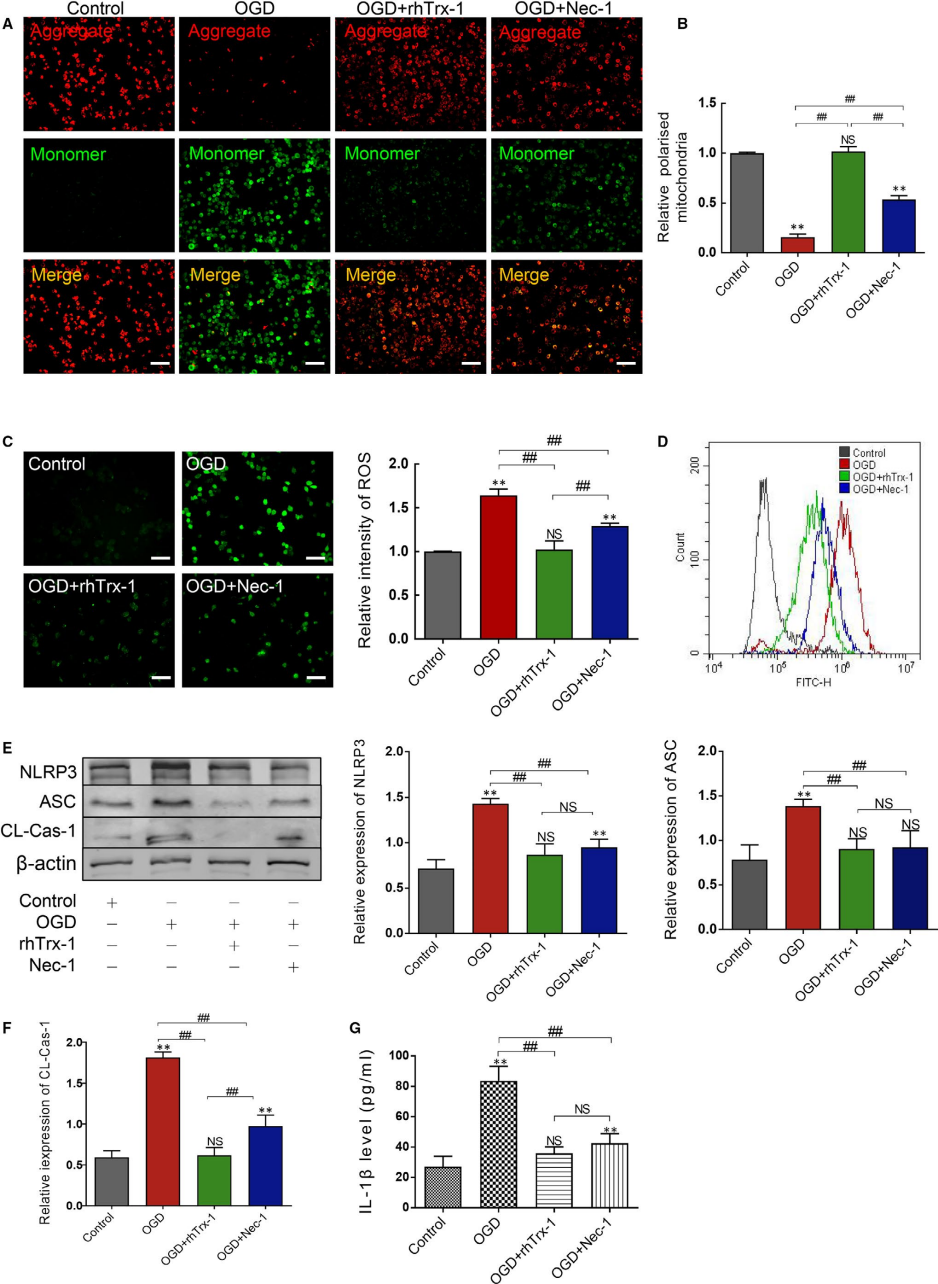
Measurement of mitochondrial membrane potential, ROS and NLRP3 inflammasome activation. A, JC‐1 staining was detected using fluorescence staining. B, The relative ratio of aggregate to monomer was analysed. C, Fluorescence intensity of ROS was analysed using fluorescence staining and D, flow cytometry. E, Western blot images of the NLRP3 inflammasome and quantitative analysis of NLRP3, ASC and F, CL‐Cas‐1 (Cleaved‐Caspase‐1) levels. G, ELISA data of IL‐1β. All scale bars represent 50 μm (n = 4 per group). **P < .01; ^##^ *P* < .01; NS, no significance

To determine the impact of rhTrx‐1 on the mitochondrial injury in ischaemic microglia, we measured the mitochondrial potential using JC‐1 staining. The mitochondrial membrane potential was dwindled (aggregate was decreased and monomer increased) following exposure to the conditions of OGD 4 hours/reoxygenation (R) 24 hours, whereas rhTrx‐1 and Nec‐1 treatment reversed the reduction of the potential (Figure 3A,B). Further analysis demonstrated that rhTrx‐1 and Nec‐1 treatment eliminated the accumulation of ROS in OGD‐induced microglia (Figure 3C,D). Finally, the inflammasome protein levels were examined to determine the activation of inflammasomes. Compared with the untreated groups, NLRP3, ASC and cleaved‐caspase‐1 levels in the rhTrx‐1 and Nec‐1 treatment groups were significantly decreased (Figure 3E,F) and the release of IL‐1β was significantly reduced as well (Figure 3G).

### 3.5 Therapeutic treatment with rhTrx‐1 regulates OGD‐induced microglia polarization and inhibits the release of inflammatory mediators

**Figure 4:**
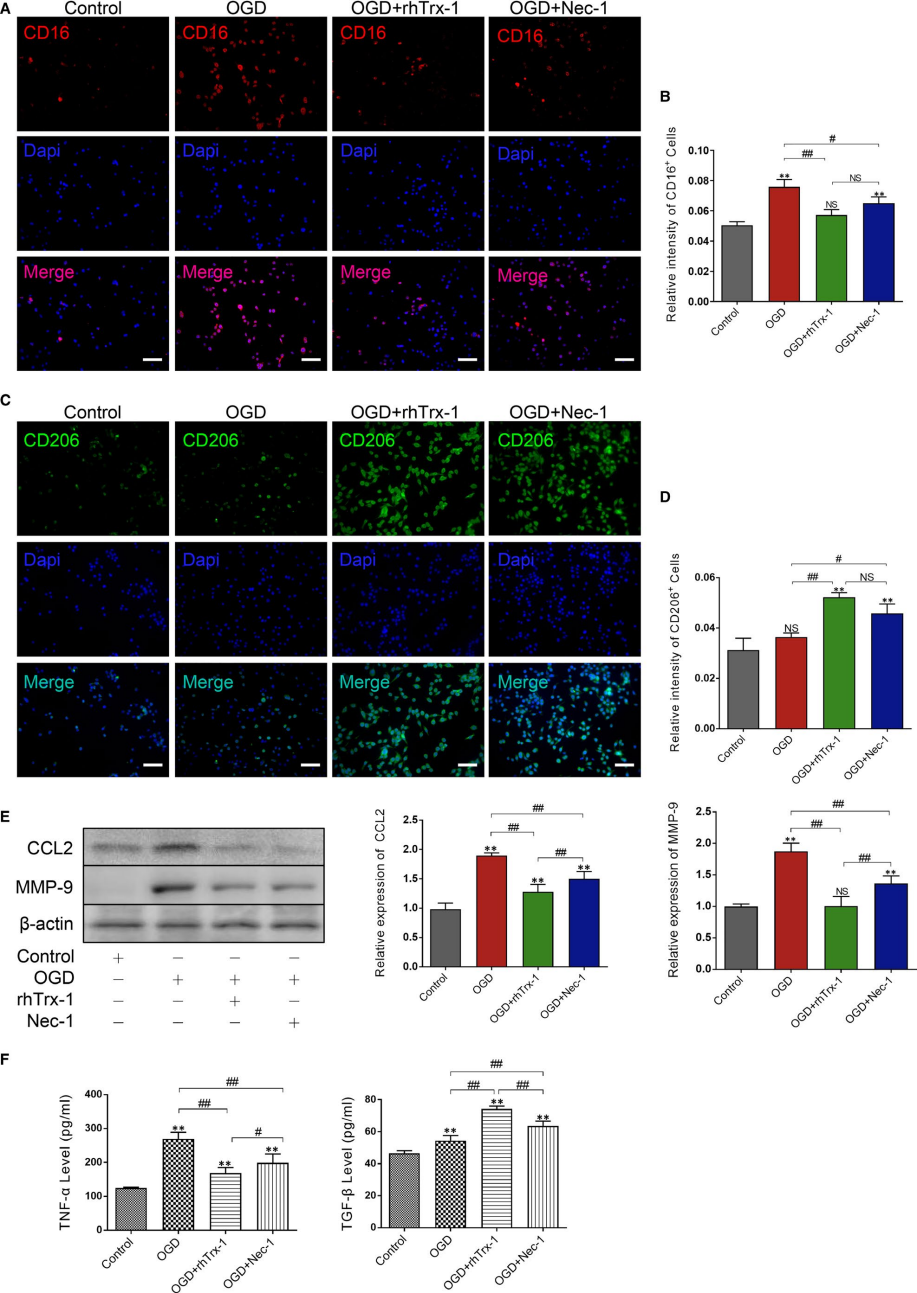
Therapeutic effects following rhTrx‐1‐mediated OGD‐induced microglia polarization causes inhibition of the release of the inflammatory mediators. A, Fluorescence staining of CD16 and Dapi. B, Quantification analysis of the fluorescence intensity of CD16+ cells. C, Fluorescence staining of CD206 and DAPI staining. D, Quantification analysis of the fluorescence intensity of CD206+ cells. E, Western blot analysis of CCL2 and MMP‐9 levels. F, ELISA data of TNF‐α and TFG‐β (n = 4 per group). Scale bar represents 50 μm. *P < .05, **P < .01; ^#^ *P* < .05, ^##^ *P* < .01; NS, no significance

To clarify the effects of rhTrx‐1 on the microglia polarization, we observed the intensity of CD16 and CD206. As demonstrated in Figures 4A,B and S2A,B, the fluorescence intensity of CD16 and CD86, representing M1‐type microglia, was enhanced in OGD and was significantly decreased following treatment with rhTrx‐1 and Nec‐1. By contrast, the fluorescence intensity of M2 microglia‐labelled CD206 was increased and treatment with rhTrx‐1 and Nec‐1 further augmented this effect (Figures 4C,D and S2C,D). Moreover, the increase in the expression of the inflammatory mediators CCL2, MMP‐9 and TNF‐α was diminished following rhTrx‐1 and Nec‐1 treatment and the expression levels of TFG‐β were increased further compared with those of the untreated group. The increased M1 microglia‐related inflammatory factors aggravate the neuronal apoptosis under ischaemic stroke.^5^ To investigate the role of inhibition of microglia RIPK1 on neuronal apoptosis following OGD, HT22 cells were co‐cultured with BV2 cells under OGD by using Anneixin V/7AAD assay detection. Results showed that the ratio of Annexin V^+^/7AAD^+^ HT22 cells under OGD was further increased compared with HT22 cells in Vehicle group (HT22 cells without co‐cultured), and rhTrx‐1 treatment decreased the ratio of Annexin V^+^/7AAD^+^ HT22 cells (Figure S2E,F).

### 3.6 Administration of rhTrx‐1 reduces acute cerebral ischaemic stroke injury

**Figure 5:**
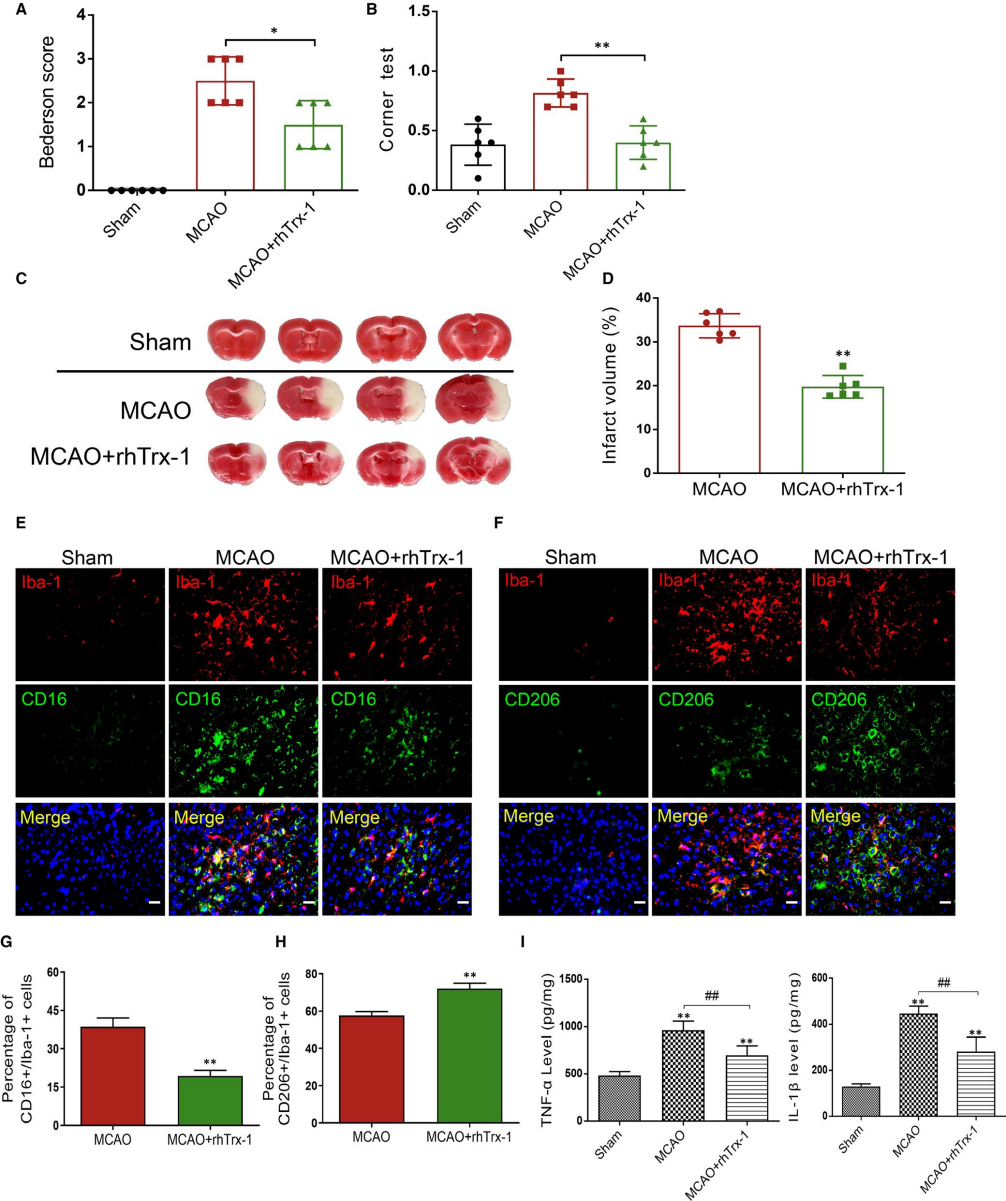
Investigation of neurological function and cerebral infarction and detection of RIPK1 levels, microglia polarization and inflammatory factors in MCAO mice. A, Quantification analysis of Bederson score. B, Quantification analysis of Corner test. C, TTC staining of mouse brain tissues. D, Quantification analysis of TTC staining (n = 6 per group). E, Fluorescence staining of Iba‐1, CD16 and DAPI. F, Fluorescence staining of Iba‐1, CD206 and DAPI. G, Quantification data of CD16+/Iba‐1+ cell numbers. H, Quantification data of CD206+/Iba‐1+ cell numbers (n = 5 per group). H, ELISA data of TNF‐α. I, ELISA data of IL‐1β detection. Scale bar represents 5 μm (n = 6 per group). *P < .05, **P < .01; ^##^ *P* < .01; NS, no significance

We examined the neurological function of mice following 24 hours of MCAO reperfusion. Berderson score and corner test results indicated that the neurological deficits of rhTrx‐1 treated MCAO mice were significantly reduced compared with those of the untreated MCAO mice (Figure 5A,B). The volume size of the cerebral infarction was detected by TTC staining, and the size of the cerebral infarction in the rhTrx‐1 treatment group was significantly smaller than that in the untreated MCAO group (Figure 5C,D). The results of the TTC staining supported the results of the neurobehavioral testing.

### 3.7 Administration of rhTrx‐1 reduced RIPK1‐mediated microglia inflammation factors release in MCAO mice

Subsequently, the numbers of CD16+/Iba‐1+ and CD206+/Iba‐1+ positive microglia were counted to estimate the polarization of M1/M2‐type microglia. Immunofluorescence staining results demonstrated that rhTrx‐1 inhibited and promoted in vivo M1‐type and M2‐type microglial activation, respectively (Figure 5E‐H). Finally, ELISA results indicated that rhTrx‐1 administration significantly reduced the release of the inflammatory mediators TNF‐α and IL‐1β (Figure 5I).

## 4 DISCUSSION

Microglial neurotoxicity is directly associated with the adverse outcome of ischaemic brain injury.^5^, ^6^, ^30^, ^31^ In the present study, we performed the MCAO and OGD experimental models to assess the RIPK1‐mediated inflammatory response in microglia and demonstrated that rhTrx‐1 could work as an inhibitor of RIPK1 to regulate the inflammatory activation of microglia following cerebral ischaemia.

RIPK1 is considered as a key regulator of innate immunity to regulate the occurrence of inflammatory response.^9^, ^32^ As a promoter of programmed cell death, RIPK1 can recruit RIPK3 to form the necrosome and finally activate the phosphorylation of MLKL to activate the necroptosis‐signalling pathway. This further causes the cells to undergo necrotic changes and increases the release of pro‐inflammatory mediators, such as MMP‐9 and TNF‐α to intensify the induction of neuroinflammation.^33^, ^34^ Huang and Fan et al indicated that Nec‐1 could inhibit the inflammatory response mediated by microglia in the retina and spinal cord by activating the necroptosis pathway.^34^, ^35^ These findings suggested that inhibition of the necroptosis pathway proteins in microglia might be beneficial to provide anti‐inflammatory effects for ischaemic stroke. Our data firstly indicated that RIPK1 levels were increased following MCAO and that RIPK1 was localized in microglia. To examine its role in ischaemic stroke‐induced microglial neuroinflammation, the expression levels of RIPK1 were subsequently assessed in vitro. The expression levels of RIPK1 were increased following the increase in the reoxygenation period in microglia. Besides, following Nec‐1 or rhTrx‐1 treatment, the induction of necroptosis by RIPK1 was significantly decreased in OGD‐induced microglia, which indicating that rhTrx‐1 exhibited similar pharmacological effects to those of Nec‐1. In accordance with these results, we further used a docking model and found that rhTrx‐1 was may directly bound to RIPK1 with Pi interactions, salt bridges and hydrogen bonds. All the findings illustrated that rhTrx‐1 could produce a marked effect in the inhibition of RIPK1.

In addition to activating necroptosis, RIPK1 can initiate apoptosis as a scaffold protein.^36^ Although it is believed that apoptosis is not involved in the release of inflammatory factors, the apoptotic microglia may indirectly aggravate the damage of cerebral ischaemic tissues and cells because of the loss of normal physiological functions. We found that rhTrx‐1 and Nec‐1 treatment reduced the activation of cleaved‐caspase‐3 following OGD in microglia, suggesting that rhTrx‐1 exhibits anti‐apoptotic effects in ischaemic microglia by inhibiting RIPK1 levels. In addition to activating caspase‐3, RIPK1 can increase the expression of pyruvate dehydrogenase complex by recruiting RIPK3, which promotes the release of ROS production in the mitochondria. And that make further efforts to increase the production of ROS in a feed forward manner.^37^, ^38^ In this way, the feedback loop promotes the accumulation of ROS, leading to mitochondrial damage and activation of mitochondrial‐mediated caspase‐3 activation.^39^ To figure out, in the present study, the production of ROS in microglia and the change in the mitochondrial membrane potential were examined and the data demonstrated that rhTrx‐1 and Nec‐1 effectively inhibited the pathological accumulation of ROS and rescued mitochondrial injury. Such protective effects may be because of the inhibition of the cascade reaction of RIPK1. Although rhTrx‐1 has demonstrated antioxidant effects and affects mitochondrial function and ROS production by regulating the function of the cell oxidative respiratory chain during treatment.^40^ However, as a result of the complexity of the mechanisms involved during oxidative stress injury, a single type of treatment for oxidative stress would be possibly unsuccessful. The present study provides evidence for the therapeutic effect of rhTrx‐1 from another perspective.

ROS accumulation was able to activate the NLRP3 inflammasome, and the activated NLRP3 inflammasome subsequently mediated neuroinflammation during ischaemic stroke by secretion IL‐1β. Deficiency of NLRP3 significantly alleviated neuroinflammation in ischaemic stroke and ischaemic injury.^41^ It has been reported that RIPK1 can trigger the activation of the NLRP3 inflammasome by disrupting the mitochondrial membrane integrity and by promoting the release of ROS, which further promotes the production and secretion of IL‐1β to the extracellular space and initiates neuroinflammation. Furthermore, the activation of RIPK3‐MLKL can directly trigger the activation of the NLRP3 inflammasome.^11^, ^42^ To investigate the role of rhTrx‐1 in RIPK1‐induced NLRP3 inflammasome activation in ischaemic microglia, we examined the levels of NLRP3, ASC and cleaved‐caspase‐1 and indicated that rhTrx‐1 ultimately reduced the release of IL‐1β, depending on the inhibition of the NLRP3 inflammasome. These results highlighted the role of rhTrx‐1 in inhibiting microglia associated‐inflammation in cerebral ischaemic stroke.

**Figure 6:**
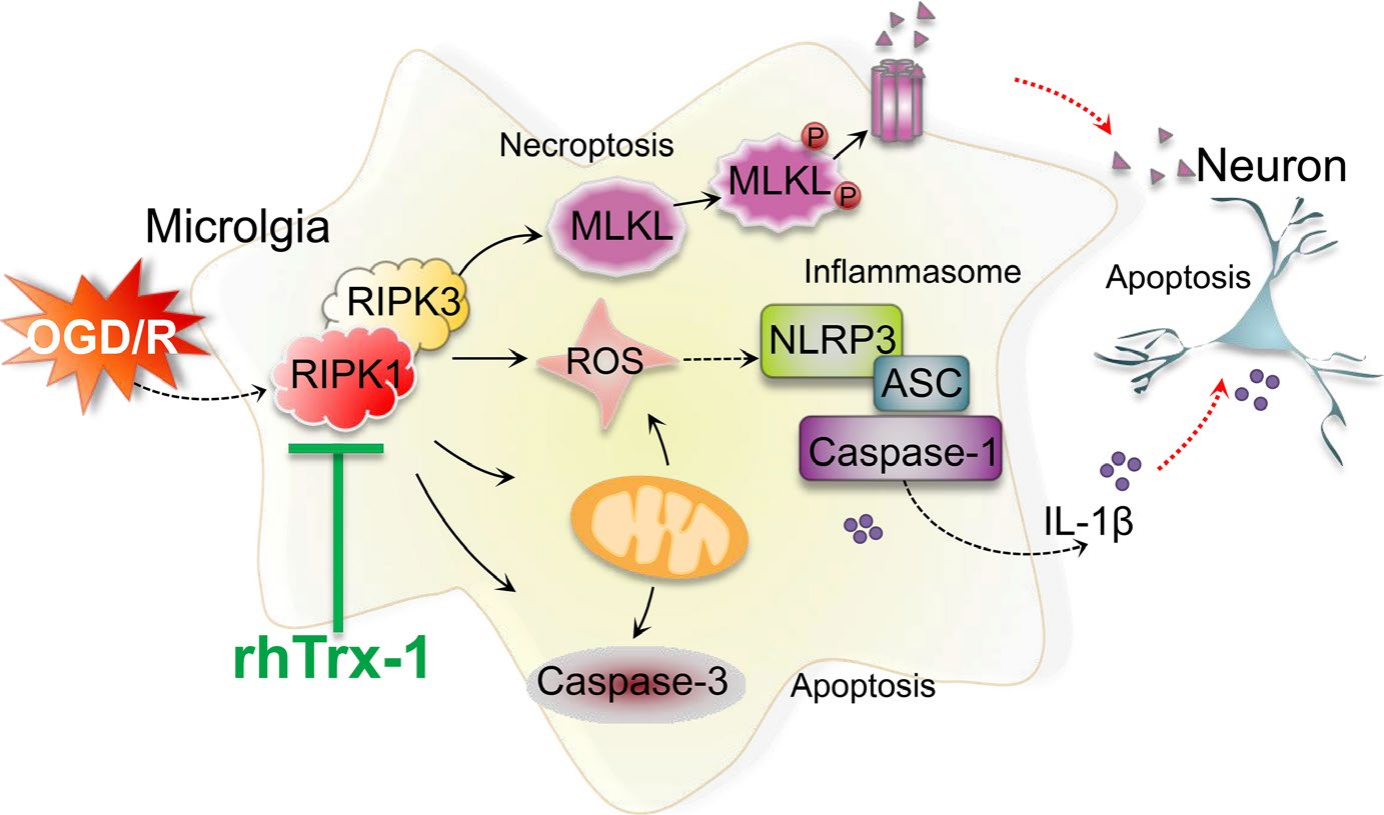
The mechanisms of rhTrx‐1 treatment on OGD‐induced microglia activation involve inhibition of RIPK1‐mediated necroptosis, apoptosis and activation of the NLRP3 inflammasome which decreased neuronal apoptosis under OGD

Following ischaemia, the activated microglia exhibit a dual function by promoting and inhibiting inflammation because of the M1/M2 polarization phenotype. M1 phenotype microglia are pro‐inflammatory, releasing inflammatory factors, while M2 phenotype is anti‐inflammatory, contributing to the repair of damaged cells. The neuroprotective effect of microglia on ischaemic stroke mainly depends on M2 phenotype microglia.^6^, ^14^, ^43^, ^44^ Our data showed that inhibition of RIPK1 expression levels significantly decreased M1 phenotype‐related molecular and inflammatory factors expression (CD16, CD86, CCL2, MMP‐9, TNF‐α and IL‐1β) following OGD reperfusion. In contrast, inhibition of RIPK1 expression significantly increased M2 phenotype‐related molecular and inflammatory factors expression (CD206 and TGF‐β). Compared with untreated OGD group, rhTrx‐1 treatment further inhibited the expression of M1 phenotype‐related molecules and increased the expression of M2 phenotype‐related molecules. These findings suggest that RIPK1 plays a role in mediating the secretion of inflammatory factors in M1 phenotypic microglia after OGD. RhTrx‐1 therapy inhibits the expression of microglial RIPK1 and regulates the expression of M1/M2 phenotype of microglia, which ultimately plays a neuroprotective role.^45^, ^46^ Besides, inhibition of inflammation during ischaemic stroke contributes to reduce ischaemic infarction.^24^, ^30^, ^41^ Based on this evidence, we further investigated the effect of rhTrx‐1 on neurological function and cerebral infarction volume. Compared with the MCAO group, rhTrx‐1 treatment alleviated the absence of neurological deficit following MCAO and effectively inhibited the volume of cerebral infarction. Similar to our results, Hattori et al have demonstrated that rhTrx‐1 was able to permeate the blood‐brain barrier and to significantly ameliorate neurological function deficit and infarct volume size.^20^ Ma et al demonstrated that rhTrx‐1 exerts an antioxidative effect against neurological dysfunction and cerebral infarction.^21^ Unlike these studies, we provided a new mechanism by which rhTrx‐1 acted as an inhibitor of RIPK1 to reduce cerebral ischaemic injury, and we found inhibition of microglia RIPK1 reduced neuronal apoptosis (Figure 6).

In conclusion, the present study demonstrated that RIPK1 plays an important role in microglia‐mediated neuroinflammatory response following cerebral ischaemia. It can regulate the conversion of microglia M1/M2 phenotype, reduce the release of inflammatory factors and ultimately reduce the occurrence of neuroinflammation by inhibiting necroptosis and the activation of the NLRP3 inflammasome. RhTrx‐1 provides neuroprotection in microglial inflammation induced by increased RIPK1 expression in ischaemic stroke.

## CONFLICT OF INTEREST

The authors declare that they have no conflict of interests.

## AUTHOR CONTRIBUTION

**Yang Jiao:** Conceptualization (lead); Data curation (equal); Formal analysis (equal); Funding acquisition (supporting); Investigation (lead); Methodology (lead); Resources (equal); Software (equal); Validation (equal); Visualization (equal); Writing‐original draft (lead); Writing‐review & editing (equal). **Jianjian Wang:** Conceptualization (equal); Project administration (equal). **Huixue Zhang:** Conceptualization (equal); Project administration (equal). **Yuze Cao:** Conceptualization (equal); Project administration (equal). **Yang Qu:** Formal analysis (equal); Investigation (equal); Methodology (equal); Validation (equal). **Siyu Huang:** Formal analysis (equal); Investigation (equal); Methodology (equal); Validation (equal). **Xiaotong Kong:** Writing‐original draft (equal); Writing‐review & editing (equal). **Chang Song:** Writing‐original draft (equal); Writing‐review & editing (equal). **Jie Li:** Formal analysis (equal). **Qian Li:** Formal analysis (equal). **Heping Ma:** Conceptualization (equal); Funding acquisition (equal); Project administration (equal). **Xiaoyu Lu:** Conceptualization (equal); Formal analysis (equal); Project administration (equal); Supervision (equal). **Lihua Wang:** Conceptualization (equal); Funding acquisition (lead); Project administration (lead); Resources (lead); Writing‐review & editing (lead).

## Supporting information

Figure S1

Figure S2

Supplementary Material

## Data Availability

The data that support the findings of this study are available from the corresponding author upon reasonable request.
