# Supplementary material for: Inhibition of microglial receptor‐interacting protein kinase 1 ameliorates neuroinflammation following cerebral ischaemic stroke
Source: J Cell Mol Med. 2020 Sep 29;24(21):12585–98. doi: 10.1111/jcmm.15820 (PMC7686994; doi:10.1111/jcmm.15820)
Supplement: Supplementary file 1 — Figure S1 [file JCMM-24-12585-s001.docx]

**Supplementary Figure 1**

**
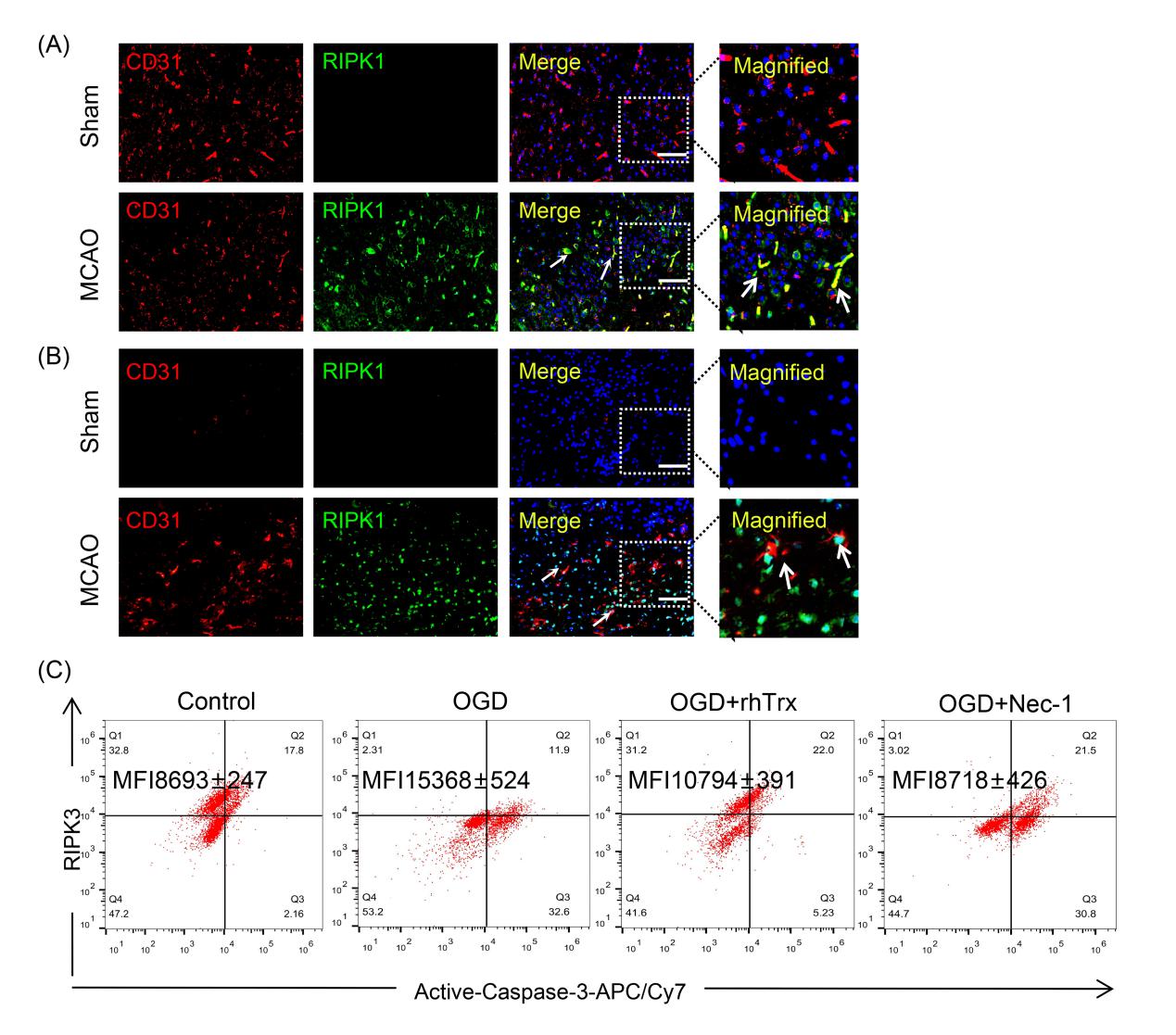
**

**Figure S1** Detection of RIPK1 level under MCAO and RIPK3 level under OGD. (A) Co-localization fluorescence staining of RIPK1 and endothelial cells (CD31) at 24 h following reperfusion in Sham and MCAO group. (B) Co-localization fluorescence staining of RIPK1 and astrocyte cells (GFAP) at 24 h following reperfusion in Sham and MCAO group. (C) Detection of the RIPK3 levels by using flow cytometry analysis.
