# Supplementary material for: Inhibition of microglial receptor‐interacting protein kinase 1 ameliorates neuroinflammation following cerebral ischaemic stroke
Source: J Cell Mol Med. 2020 Sep 29;24(21):12585–98. doi: 10.1111/jcmm.15820 (PMC7686994; doi:10.1111/jcmm.15820)
Supplement: Supplementary file 2 — Figure S2 [file JCMM-24-12585-s002.docx]

**Supplementary Figure 2**


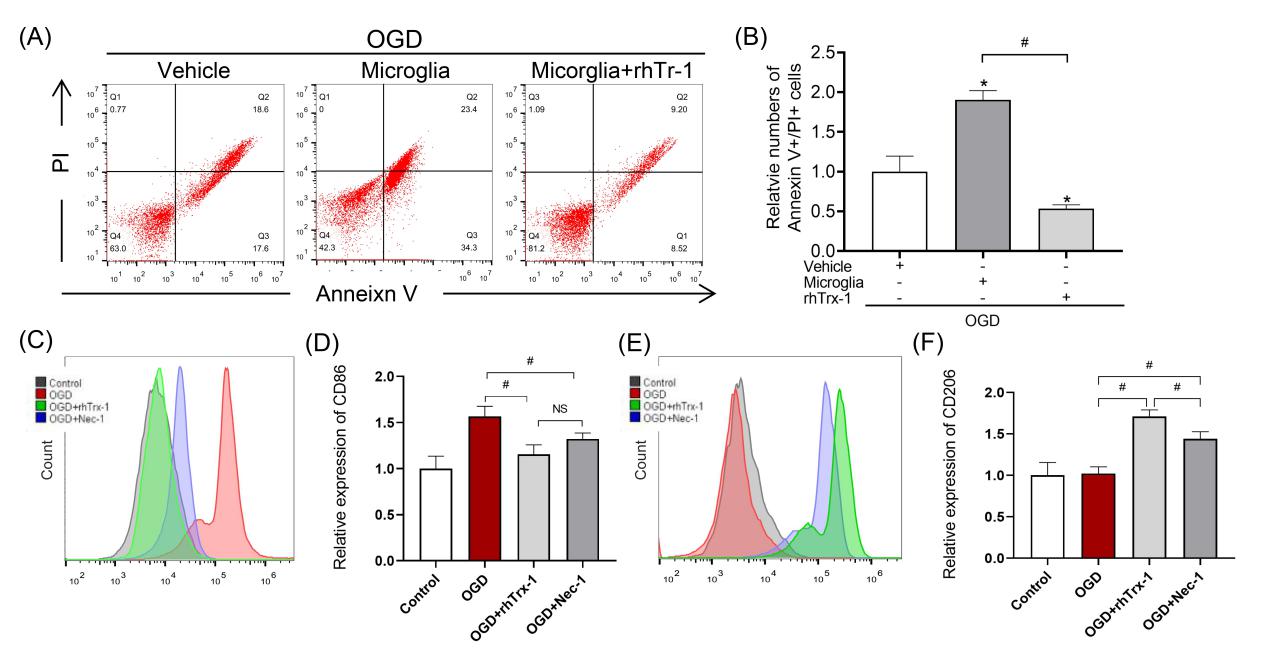


**Figure S2** Detection of microglia polarization and neuronal apoptsis in vitro. (A, B) Detection and quantification analysis of the CD86 levels by using flow cytometry analysis. (C, D) Detection and quantification analysis of the CD206 levels by using flow cytometry analysis. (F, F) Detection and quantification analysis of the AnexinV/7AAD positive HT22 cells by using flow cytometry analysis.
