# Supplementary material for: Inhibition of microglial receptor‐interacting protein kinase 1 ameliorates neuroinflammation following cerebral ischaemic stroke
Source: J Cell Mol Med. 2020 Sep 29;24(21):12585–98. doi: 10.1111/jcmm.15820 (PMC7686994; doi:10.1111/jcmm.15820)
Supplement: Supplementary file 3 — Supplementary Material [file JCMM-24-12585-s003.docx]

**Mehtods**

**Immunofluorescence staining**

Briefly, in vivo immunofluorescence was assessed using frozen slices that were washed with PBS for 3 times and subsequently blocked with 5% BSA at room temperature for 1 h. The anti-RIPK1, anti-CD31, and anti-GFAP primary antibodies were incubated with the slices at 4°C overnight. The second day slices were incubated with the secondary antibodies (Alexa Fluor 555-conjugated donkey-anti rabbit and Alexa Fluor 488-conjugated donkey-anti mouse. The slices were imaged with fluorescence microscopy (ZEISS, Jena, Germany).

**Flow cytometry**

Harvested cells were washed in PBS/BSA and permeabilised in

0.25% Triton X-100 for 15 min at RT. After washed, cells were then stained with anti-RIP3-PE (Santa Cruz, USA)and anti-active caspase-3-Alexa 647 (BD Biosciences, USA) for 20 min at RT. The cells were finally analyzed using a Beckman CytoFLEX flow cytometer (CA, USA).
